# The Recurrence of Tracheobronchial Papillomatosis Following Prostate Cancer Diagnosis

**DOI:** 10.7759/cureus.74450

**Published:** 2024-11-25

**Authors:** Tilemahos Iakovakis, Fotios Drakopanagiotakis, Efterpi Tirikidou, Alexandra Giatromanolaki, Mary Kouroupi, Vasiliki E Georgakopoulou, Paschalis Steiropoulos

**Affiliations:** 1 Department of Respiratory Medicine, School of Medicine, Democritus University of Thrace, Alexandroupolis, GRC; 2 Department of Pathology, University General Hospital of Alexandroupolis, Alexandroupolis, GRC; 3 Department of Pathophysiology/Pulmonology, Laiko General Hospital, Athens, GRC

**Keywords:** biopsy, bronchoscopy, cough, prostate cancer, recurrent respiratory papillomatosis

## Abstract

Recurrent respiratory papillomatosis (RRP) is a challenging disease to manage, due to its highly recurring nature and the lack of a definitive treatment. It is characterized by the presence of benign papillomatous lesions caused by the human papillomavirus (HPV), which can pose a threat to the patient’s airway patency and restrict their breathing ability. We present the case of a 64-year-old patient with a history of papillomas in the trachea and bronchi, treated with endobronchial cryotherapy. However, tracheal and bronchial papillomas recurred 10 years after the initial treatment and two years after the diagnosis of prostate cancer and hormonal therapy. We also engage in a discussion of the clinical and radiological features of RRP.

## Introduction

Recurrent respiratory papillomatosis (RRP) is characterized by the occurrence of benign papillomatous lesions caused by the human papillomavirus (HPV), which usually manifests in the larynx, but can be found anywhere in the aerodigestive tract, like the trachea, nasopharynx or in the pulmonary parenchyma. It is more commonly caused by the subtypes HPV-6 and HPV-11, two types also involved in condylomata lata [1-3]. The main symptoms are nonspecific and may include chronic cough, progressive hoarseness, stridor, dyspnea, wheezing, and acute respiratory distress [2]. RRP can be threatening to the patient’s airway patency. It is a challenging disease due to its high recurrence rate and can severely affect the patient’s quality of life [4]. Many patients who suffer from this disease require multiple surgeries, while in others, the disease spontaneously regresses. Each child diagnosed with RRP requires on average 19.7 procedures in their lifetime and a mean of 4.4 surgeries per year [5].

Tracheal papillomatosis occurs either after laryngeal papillomatosis spreads to the trachea, or isolated on the trachea [6]. Some studies indicate a high incidence of tracheal involvement, as high as 29% [7], in patients with laryngeal papillomatosis. Tracheobronchial involvement significantly complicates the progression of the disease, and the probability of it occurring seems to dramatically increase after tracheostomy [7,8].

## Case presentation

**Figure 1:**
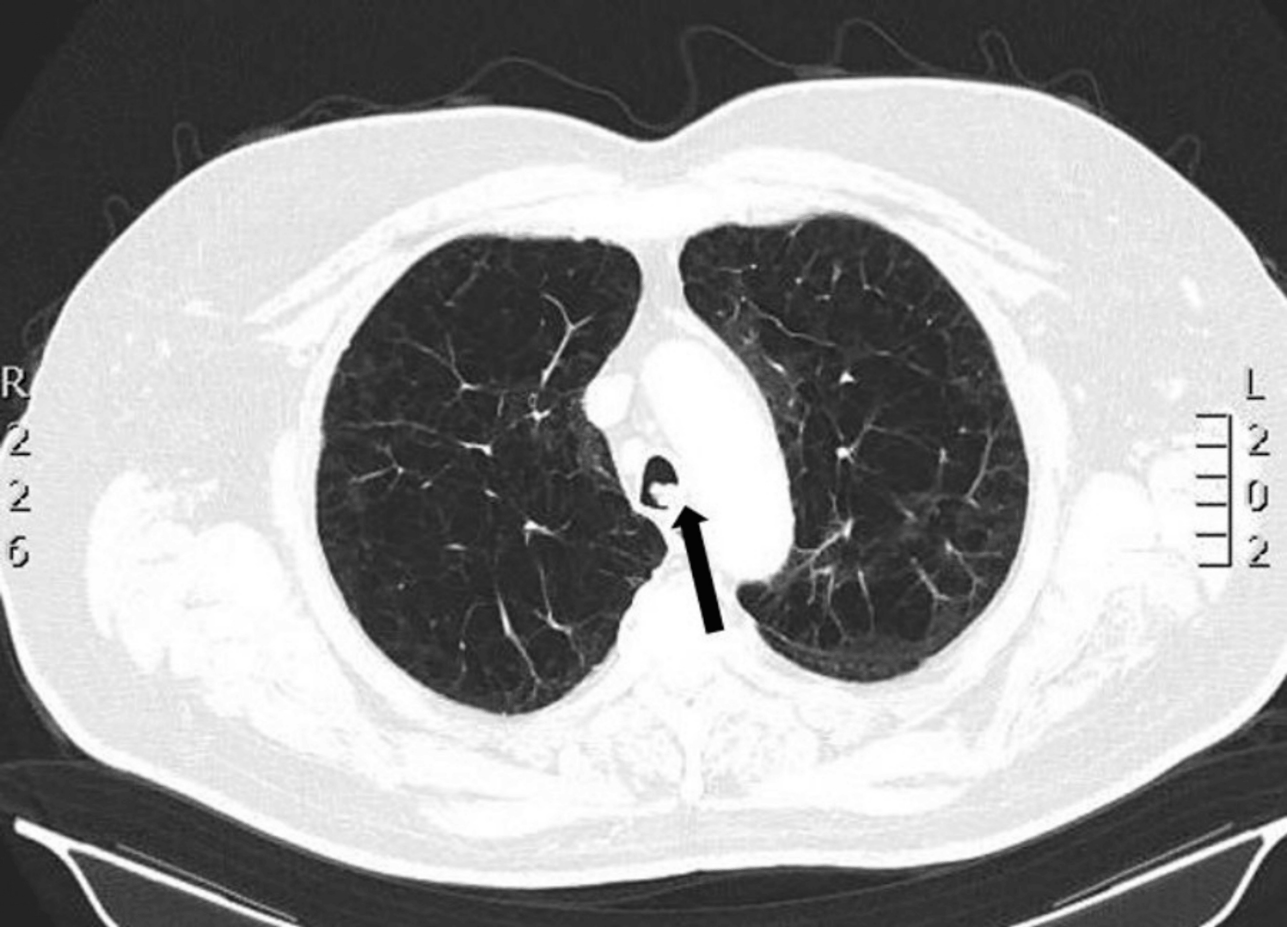
Chest CT (lung window) The image showed papillomas (black arrow) on the left wall of the trachea and significant emphysema in the lung parenchyma CT: computed tomography

**Figure 2:**
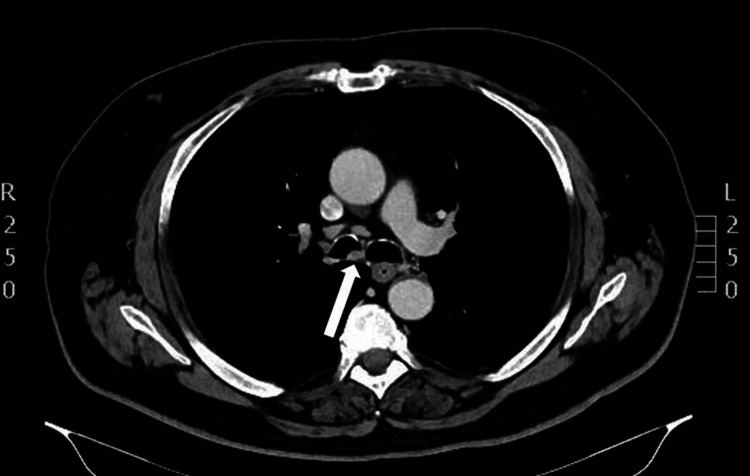
Chest CT (mediastinal window) The image showed papillomas (white arrow) in the right bronchus CT: computed tomography

A 65-year-old male patient with symptoms of productive cough and occasional hemoptysis (blood-streaked mucus) presented to our hospital for further evaluation. He had been diagnosed with tracheal papillomas and received endobronchial cryotherapy 10 years ago. He had also been diagnosed with prostate cancer two years prior and was under treatment with bicalutamide and leuprorelin. A chest CT performed at the time of prostate cancer diagnosis had not shown any lung abnormalities. He was also suffering from COPD and undergoing treatment with inhaled fluticasone/umeclidinium/vilanterol. We performed a thoracic CT scan, which showed lesions on the trachea and right bronchi (Figures 1-2).

**Figure 3:**
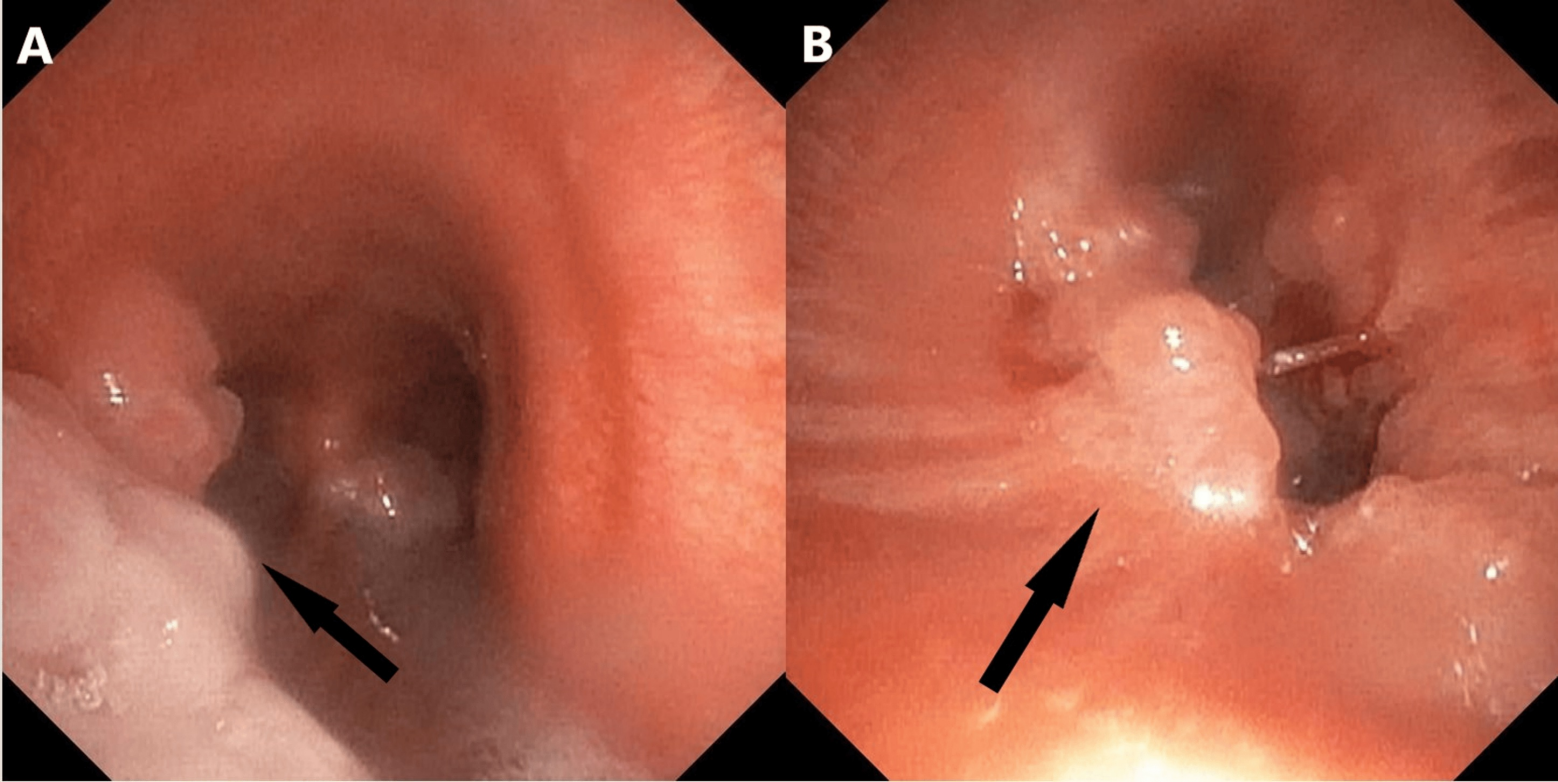
Bronchoscopic examination A: Papillomatous lesions of the trachea (black arrow). B) Papillomatous lesions of the right main bronchus (black arrow)

A bronchoscopy was performed, which showed lesions in the trachea and right main bronchus, as well as lesions in the left bronchus. Specifically, there was a 30% stenosis of the trachea by lesions stemming from the left tracheal wall, and there were lesions at the middle of the left main bronchus. In the right bronchial tree, exophytic lesions were found in the proximal right main bronchus, and the bronchus intermedius (Figure 3).

**Figure 4:**
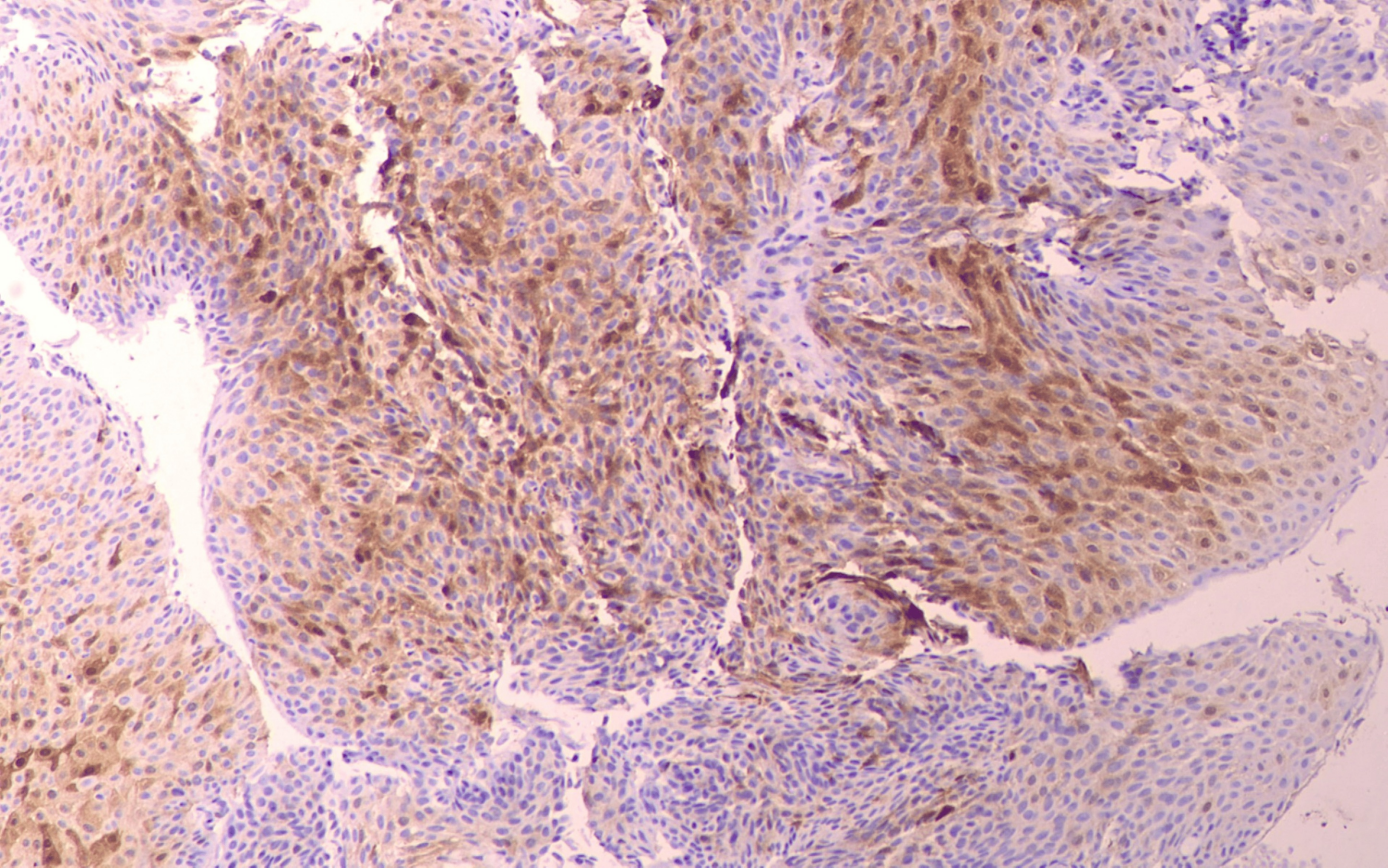
Histological examination of specimens obtained by bronchoscopy Immunohistochemistry revealed p16 (+)

The extent of these lesions found was more extensive compared to the ones reported 10 years ago, particularly regarding the left main bronchus. During the bronchoscopy, biopsies of the lesions were taken. Histological examination confirmed the diagnosis of papillomas and also showed moderate dysplasia of the squamous epithelium, which had not been present in the previous tests 10 years ago. The immunochemical tests came back positive for p16, a marker that indicates the existence of the HPV (Figure 4).

Further treatment with endobronchial cryotherapy was planned. Endobronchial cryotherapy is a cryosurgical technique, that allows freezing tissue in extremely low temperatures for destruction, thereby effectively managing central airway obstruction, mainly due to malignant tumors. A cryoprobe is inserted through the bronchoscope and pathological tissue is frozen at -80 ºC using nitrous oxide or liquid nitrogen [8].

## Discussion

At the time of our patient’s admission to the hospital, it was not clear whether the lesions in the trachea and bronchus were due to papillomatosis. The differential diagnosis for multiple tracheal masses included direct invasion of the trachea from tumors of the lung, esophagus, or larynx; primary tracheal tumors; multifocal neoplasms; and amyloidosis [9].

One interesting feature of RRP is its bimodal age distribution: it is categorized into juvenile-onset RRP (JORRP) and adult-onset RRP (AORRP) based on it being diagnosed before or after 12 years of age, respectively. JORRP is diagnosed most commonly reported between two and four years of age and AORRP between the second and fourth decade of a patient’s life [5]. JORRP is thought to be caused by infection of the child transmitted vertically from its mother, and the chances of transmission are increased when the mother has a recent HPV anogenital infection, longer duration of labor, and is giving birth for the first time. In adults, it is believed that oral sex can transmit the virus and cause RRP, but the presence of the virus in the mucosa of the airway by itself is not enough to cause the infection [3].

RRP lesions are mainly benign. However, some patients (1% in children and 3-7% in adults) will present with carcinoma, particularly squamous cell carcinoma (SCC), emerging from these lesions; hence, a biopsy is recommended when diagnosing RRP [1]. In our patient, the papillomatosis had significantly spread to the bronchial tree, compared to the ones 10 years ago, although the patient had undergone therapeutic endobronchial cryotherapy and a CT scan of the lung two years ago had not shown any abnormalities. Given that the disease had spread and moderate dysplasia of the mucosa was found, there was a need for closer monitoring of the patient’s condition even after any treatment, since pulmonary RRP is more likely (even 32 times more than regular RRP) to undergo malignant transformation and has an overall worse prognosis than regular RRP [10].

Reports have implicated HPV in the pathogenesis of prostate cancer [11,12]. It raises the question as to whether both these diseases could develop from the same virus, and if the development of prostate cancer is linked to the reappearance of papillomas. Additionally, the treatment for prostate cancer or the hormone imbalance from the cancer itself might affect the balance of the immune system and lead to the reawakening of the virus in the respiratory epithelium, as well as the dysplasia of the epithelium [13]. Leuprorelin is an LHRH analog, which desensitizes the frontal lobe of the pituitary gland to the hormone’s signal, causing a suppression of the production of LH and FSH. These hormones play a key role in the body’s function in both genders, and LH stimulates the production of testosterone in the testes. Leuprorelin can disrupt immunological pathways and affect the immune system’s function [14]. Also, leuprorelin use is associated with upper respiratory tract infections and herpes simplex reactivation [15]. However, there is scarce data supporting HPV reactivation due to leuprorelin or prostate cancer. It cannot be excluded whether reactivation of HPV after prostate cancer diagnosis and treatment is a mere temporal coincidence. Further research is required to clarify the role of HPV in the development of prostate cancer and if patients suffering from one disease are more at risk of developing the other.

The patient´s productive cough could be easily attributed to the presence of COPD. However, hemoptysis and change in cough characteristics, particularly in patients with a history of malignancy are "red flags", warrant further evaluation. It has also been reported that RRP can be misdiagnosed as asthma or COPD with persistent cough and wheezing as overlapping symptoms of the conditions. Therefore, these two conditions should be included in the differential diagnosis [16]. Also, COPD can impact the epithelium of the respiratory tract, making it more susceptible to infections. The irritants that contribute to the development of COPD, as well as the inflammation in COPD and recurring infections, can all weaken the epithelium’s defenses and promote premature cell senescence [17].

There is no definitive treatment for RPP, and the current options include excision of the lesions, with 20% of patients requiring adjuvant therapies to support treatment. Traditionally the techniques that have been used include potassium titanyl phosphate (KTP) lasers, microdebriders, CO_2_ lasers, or cold steel lasers. Adjuvant therapies like interferon, HPV vaccine, bevacizumab, cidofovir, celecoxib, and others, are used when the disease is difficult to manage by interventional methods alone [18]. These options can increase the intervals between interventions. Interferon was one of the earliest adjuvant therapies for RRP, but bevacizumab and cidofovir have also shown promising results recently. HPV vaccine holds the potential to be a treatment option (as well as one for prevention), with several studies showing promising results [19,20].

## Conclusions

This report highlights the importance of continuous, lifelong vigilance when treating patients with RRP. Even after many years without the growth of lesions, the epithelium is susceptible to developing papillomatosis again. As mentioned above, close follow-up is indicated in patients with extensive tracheobronchial disease, due to the higher risk of malignancy. Further research is needed to determine the relationship between prostate cancer and HPV. It is important to consider the changes in the epithelium’s susceptibility when a patient’s immune system is disrupted. Comorbid asthma or COPD with RRP also poses some challenges and should be taken into account, especially in older patients.
